# Supplementary figures and images for: Aquatic Pollution and Risks to Biodiversity: The Example of Cocaine Effects on the Ovaries of Anguilla anguilla
Source: Animals (Basel). 2022 Jul 10;12(14):1766. doi: 10.3390/ani12141766 (PMC9312106; doi:10.3390/ani12141766)

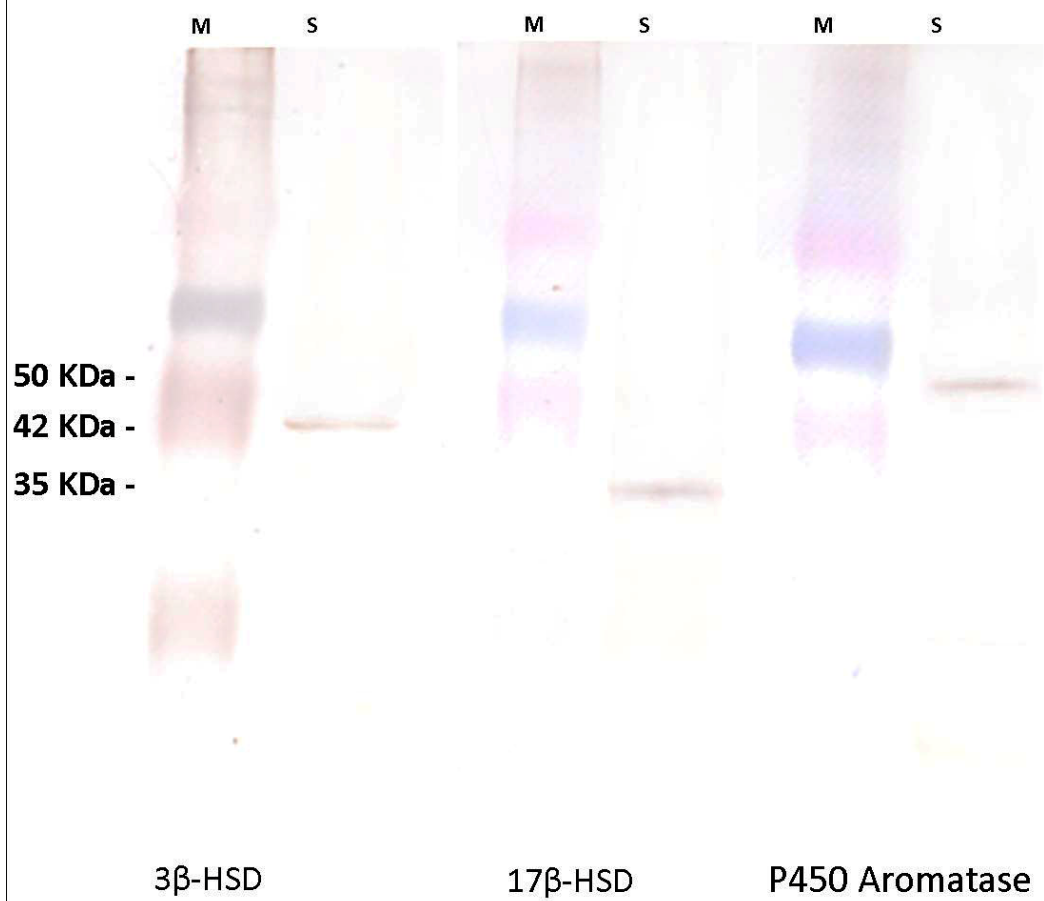

**Figure S1.** Immunoblots on *Anguilla anguilla* proteins.

Supplement: Supplementary file 1 [file animals-12-01766-s001.zip › animals-1794589-supplementary.pdf]
